# Class Collective Efficacy and Class Size as Moderators of the Relationship between Junior Middle School Students’ Externalizing Behavior and Academic Engagement: A Multilevel Study

**DOI:** 10.3389/fpsyg.2017.01219

**Published:** 2017-07-18

**Authors:** Yu Tian, Yulong Bian, Piguo Han, Fengqiang Gao, Peng Wang

**Affiliations:** ^1^Department of Psychology, Shandong Normal University Jinan, China; ^2^Department of Computer Science and Technology, Shandong University Jinan, China

**Keywords:** class collective efficacy, class size, externalizing behavior, academic engagement, multilevel study

## Abstract

This study examined the relationship between externalizing behavior and academic engagement, and tested the possibility of class collective efficacy and class size moderating this relationship. Data were collected from 28 Chinese classrooms (*N* = 1034 students; grades 7, 8, and 9) with student reports. Hierarchical linear modeling was used to test all hypotheses and results revealed a negative relationship between externalizing behavior and academic engagement; class collective efficacy was also significantly related to academic engagement. Additionally, class collective efficacy and class size moderated the relationship between externalizing behavior and academic engagement: For students in a class with high collective efficacy or small size (≤30 students), the relationship between externalizing behavior and academic engagement was weaker than for those in a class with low collective efficacy or large size (≥43 students). Results are discussed considering self-regulatory mechanisms and social environment theory, with possible implications for teachers of students’ learning provided.

## Introduction

Engaged students are characterized by vigor, dedication, and absorption (Schaufeli et al., 2002; Schaufeli and Bakker, 2004). As described by Kaplan et al. (1997), “They are attentive and participate in class discussions, exert effort in class activities, and exhibit interest and motivation in learning." By contrast, disengaged students are disruptive, are less likely to aspire to higher educational goals, have lower grades, and are more likely to drop out of school." Student engagement is vital to academic achievement (Furrer and Skinner, 2003; Reyes and Brackett, 2012; Moller et al., 2014), which is associated with lower rates of negative outcomes such as teen pregnancy, welfare dependency, and criminal behavior, as well as higher levels of positive outcomes including employment stability and lifetime income (Johnson et al., 2006). Thus, this reality highlights the need for teachers to clearly identify the factors associated with academic engagement.

### Relationship between Externalizing Behavior and Academic Engagement

Previous studies have found that students with externalizing behavior tended to be hyperactive, impulsive, and aggressive, performing poorer in school than other students do (Abikoff et al., 2002; Junod et al., 2006). As described by Rothbart and Bates (1998), “externalizing behavior was associated with self-regulation difficulties, meaning that a person had difficulty focusing or shifting attention, demonstrating persistence on tasks, activating or inhibiting behavior, and responding adaptively to novel situations.” Aforementioned deficits in self-regulation had a profound effect on appropriately engaging in academic tasks (Baker et al., 2008). In addition, “students who exhibited externalizing behavior often lacked the necessary skills to meet the academic and behavioral demands of the typical classroom, and they often found various instructional and academic task demands aversive, which led to less student engagement and more disruptive and off-task behavior” (Baker et al., 2008). Recently, Johnson et al. (2006) used hierarchical linear growth curve analysis and determined that externalizing behaviors negatively related to academic engagement across time. Furthermore, Obradović et al. (2010) replicated similar effects in a longitudinal study. The aforementioned studies have indicated that externalizing behavior was a negative individual factor associated with academic engagement; therefore, it is imperative for teachers to understand how to increase the academic engagement of students with externalizing behavior.

### The Relationships among Class Collective Efficacy, Externalizing Behavior and Academic Engagement

Engagement behaviors were a product of the interaction between the class environment and the individual (Finn and Rock, 1997), and class settings have effects on student behavior (Baker et al., 2008; Beattie and Thiele, 2016). However, “almost all existing studies have derived students’ within-class normative standings on certain variables that are analyzed independently of these class variables. Consequently, much of the between-class variation has been uncount left to confound the individual-level associations” (Reyes and Brackett, 2012). Previous studies have shown that classroom settings affect students’ engagement behaviors, such as collective pedagogical teacher culture (Moller et al., 2014), instructional setting (Baker et al., 2008), the teacher-student relationship (Klem and Connell, 2004), the collective efficacy of the teacher (Wan and Kates, 2010), and the relationship of peers (Furrer and Skinner, 2003). However, to the extent that the setting varies across classes and influences students’ behaviors, individual behaviors carry different relationships across class settings (Reyes and Brackett, 2012; Beattie and Thiele, 2016). Thus, developing a more comprehensive understanding of the relationship between class settings and individual behaviors is necessary to determine how specific class settings affect student academic engagement.

With the emergence of positive psychological study, people are interested in the great positive forces of human beings (e.g., self efficacy). Some studies have emphasized how students are active learning participators, self-regulating to change their own behaviors (Bandura, 1977, Bandura, 1994/1998; Clark, 2012). In particular, Bandura (1977) argued that “perceived efficacy beliefs are a real key mechanism for behavioral change in individuals, and that these perceived beliefs are not only related to themselves, in self-efficacy, but also to the group, in collective efficacy.” However, the aggregation of each individual’s self-efficacy was an essential factor of collective efficacy (Bandura, 1977). Thus, the positive force of human beings would be influenced by collective efficacy, particularly in students who spend most of their time in interacting or studying with teachers or classmates in a fixed class. Therefore, this led us to believe that the collective efficacy of classes could influence academic engagement, and that a different relationship would exist between externalizing behavior and academic engagement across class settings. However, a previous study revealed that “a talented person with extremely high self-efficacy could have low collective efficacy if he or she could not cooperate effectively with other group members” (Clark, 2012). Lent et al. (2006) reported that “collective efficacy was an effective predictor of team performance where outcomes depended on group cohesiveness and the aggregation of team members’ beliefs of self-efficacy.” Thus, collective efficacy involved not simply an aggregation of each individual’s self-efficacy, but also class members’ judgments of the class’s cohesiveness as a whole.

Existing studies have found that students with higher self-efficacy had a positive relationship with academic engagement, and this self-efficacy is defined as a student’s beliefs in his or her personal capabilities in performing learning tasks (Bandura, 1977, Bandura, 1994/1998; Clark, 2012). Bandura (1977; 1994/1998) has used self-regulation to elaborate the mechanisms of self-efficacy; self-regulation was referred to as “the processes, internal and/or transactional, that enable an individual to guide his or her goal-directed activities over time and across changing circumstances.” He argued that “self-regulated learning arose where there were strong perceptions of self-efficacy, and students who believed that they were capable learners were ready to assess their own work, identify their current strengths and weaknesses, and regulate themselves in the next steps, which would in turn help them develop a set of academic engagement behaviors (e.g., seek help from adults, manage time, and engage in peer learning).” However, externalizing behavior was strongly related to self-regulation difficulties (Howse et al., 2003), which would lead students to become involved in disruptive non-academic behavior rather than appropriately engaging in academic tasks (Abikoff et al., 2002; Junod et al., 2006). Thus, self-efficacy may be a beneficial moderator of the relationship between externalizing behavior and academic engagement. However, the self-efficacy of students was different; in turn, the aggregation of each class’s self-efficacy (collective efficacy) was also different. Therefore, this led us to firmly believe that the relationship between externalizing behavior and academic engagement differs across class settings.

“The classroom is a primary microsetting in which students interact with one another. The quality of social and emotional interactions in the classroom among students may influence classroom emotional states” (Ryan and Patrick, 2001; Pianta et al., 2008). Students from classrooms characterized by positive emotional states had greater emotional connection and respect for cooperation, regularly expressing warmth toward other students, which in turn increased academic engagement (Obradović et al., 2010; Reyes and Brackett, 2012). Previous studies have confirmed that members of high cohesiveness groups tend to experience more positive emotional states (Kidwell et al., 1997). “Positive emotions help students envision goals and challenges and open their minds to positive thoughts, and they may thus lead students to have greater engagement regarding their studies and exhibit greater task persistence in the face of challenges” (Jex and Bliese, 1999). Thus, we contend that more engagement behavior would exist in a high cohesiveness class (Schmuck, 1963). Moreover, cohesiveness afforded classes with resources that they could draw upon as a buffer against the disruptive effects of externalizing behavior, and members could consequently rely upon one another to share learning intentions, as well as conduct collaborative learning and effective discussion (Patrick et al., 2007). By contrast, “for low cohesiveness groups, the general lack of shared commitment to learning tasks and low interpersonal attraction between class members yield fewer available class resources for managing the disruptive effects of externalizing behavior, because the negative emotional states and weak group identity limit the degree to which group members help one another” (Kidwell et al., 1997), and this engenders less engagement behavior. Therefore, the aforementioned studies further led us to believe that a different relationship between externalizing behavior and academic engagement across class settings.

### Class Size as a Moderator of the Relationship between Externalizing Behavior and Academic Engagement

Prior studies have found that larger college classes were associated with lower student achievement, attendance, and participation (Fassinger, 1995; Arias and Walker, 2004). To determine the functionary mechanism of class size, Beattie and Thiele (2016) examined the association between class size and academic engagement. The result showed that larger classes hindered a key type of beneficial student engagement—student interactions concerning academic and career matters with professors and peers across campus settings. The reason for this may be that “when students are exposed to more peers in large classes, negative peer influence becomes greater, because the likelihood of observational learning, reinforcement learning, and peer-contagion processes increases” (Dishion and Dodge, 2005). Furthermore, social-learning theory contends that “in larger groups, children often learn antisocial behavior from peers, which might in turn lead to diminished academic engagement” (Laird et al., 2001).

As described by Skalická et al. (2015), “in a smaller preschool class, teacher-child closeness exerts a greater protective effect on future behavior problems, compared with a larger class. Young children depend on adults to be told and shown appropriate behavior and also to have their behavior monitored and corrected when necessary.” Specifically, students in smaller classes have more chances to be guided by teachers, and they would spend more time interacting with adults rather than peers, compared with larger classes (Dunn, 1993). This also reduces the chance of students learning externalizing behavior from peers. In addition, a smaller class size might impose less strain on teachers, thus providing them with more opportunities to provide emotional support and appropriate responses to students’ externalizing behavior, which might reduce academic engagement. However, to our knowledge, no prior study has systematically examined the link between junior middle school class size and academic engagement, and we expected class size variation to particularly influence the relationship between externalizing behavior and academic engagement. Therefore, in the present study, we also sought to evaluate the association between class size and individual behaviors.

### Summary of the Study

In summary, our purpose was to understand how these class settings influence student engagement behaviors and to provide teachers with possible implications for more effective academic interventions for students with externalizing behavior. Therefore, the present study investigated the influence of both externalizing behavior and class settings on students’ academic engagement. Furthermore, this study examined whether class collective efficacy and class size were moderators of the relationship between externalizing behavior and academic engagement. Following the steps in moderation (Hofmann and Gavin, 1998; Reyes and Brackett, 2012), we tested the following hypotheses:

**Hypothesis 1:** Externalizing behavior has a negative main effect on academic engagement individually.
**Hypothesis 2:** Class collective efficacy has a positive main effect on academic engagement.
**Hypothesis 3:** Class size has a negative main effect on academic engagement.
**Hypothesis 4:** The relationship between externalizing behavior and academic engagement is negatively moderated by class collective efficacy such that the relationship between externalizing behavior and academic engagement will be weaker when in a class with high collective efficacy.
**Hypothesis 5:** The relationship between externalizing behavior and academic engagement is positively moderated by class size such that the relationship between externalizing behavior and academic engagement will be weaker when in a small class.

## Materials and Methods

### Sample and Method

The present study was conducted in accordance with the 1964 Helsinki declaration and its later amendments or comparable ethical standards, with the approval of the Human Research Ethics Committee of Shandong Normal University. The final sample consisted of 1034 junior middle school students from 28 classes. Among the 28 classes, 29% were Grade 3 and 40% were Grade 2 and 31% Grade 1 (respectively, equivalent to the American Grades 9, 8, and 7). The age of the students (*M* = 15.08 years; *SD* = 1.23) ranged from 13 (12%) to 17 (10%). Female students constituted 53% of the sample. In the present sample, the junior middle school class size was between 26 and 47 students and the average class size was 36.93 (*SD* = 6.31).

**FIGURE 1:**
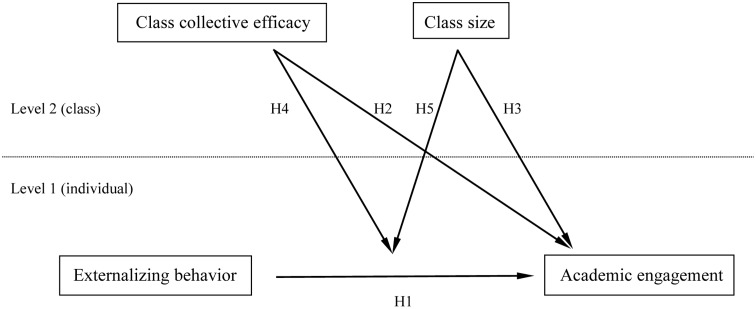
Hypothesized relationships.

**Figure 1** provides a graphical depiction of the relationships specified in the hypotheses.

### Measures

#### Externalizing Behavior

Externalizing behavior was measured by a revised Child Behavior Checklist-Student Survey (CBCL-S; Chen et al., 2009) under Chinese culture. A 20-item measure was used, and it entailed students rating the frequency of externalizing behaviors on a 6-point Likert scale ranging from 1 (*never*) to 6 (*often*). Externalizing behavior was measured by aggression behavior (10-items) and delinquency behavior (10-items) two factors. In the current sample, aggression behavior (e.g., physical attack on others and threatening others) and delinquency behavior (e.g., high online activity, drinking, or smoking) items were summed to form the externalizing score. The α coefficients for the total scale, aggression subscale, and delinquency subscale were 0.95, 0.89, and 0.97, respectively. In addition, the results of a confirmatory factor analysis using Mplus 7.0 were as follows: χ^2^*/df* = 2.46, *p* < 0.001; comparative fit index (CFI) = 0.93; non-normed fit index (NNFI) = 0.94; root mean square error of approximation (RMSEA) = 0.059; standardized root mean square residual (SRMR) = 0.05.

### Academic Engagement

We assessed academic engagement by using the Utrecht Work Engagement Scale-Student Survey (UWES-S; Schaufeli et al., 2002) consisting of 17-items. Zhang et al. (2008) confirmed that the Work Engagement Scale can measure the academic engagement of middle school students in China. Academic engagement was measured by the three factors of vigor (6-items), dedication (5-items), and absorption (6-items). An example of a vigor item is “I am willing to invest effort, and persist in the face of difficulties,” dedication “I have a sense of enthusiasm with regard to my study,” and absorption “I am fully focused on my study tasks and feel like time is flying.” Each student rated his or her agreement with the items on a 6-point Likert scale ranging from 1 (*strongly disagree*) to 6 (*strongly agree*). In this study, the α coefficients for the total scale, vigor subscale, dedication subscale, and absorption subscale were 0.92, 0.87, 0.95, and 0.91, respectively. Moreover, the results of the confirmatory factor analysis were as follows: χ^2^/*df* = 3.41, *p* < 0.001; CFI = 0.93; NNFI = 0.95; RMSEA = 0.059; SRMR = 0.04.

### Class Collective Efficacy

Class collective efficacy was measured using the Collective Efficacy Scale for Middle School Students in China (CEC-S; Chen, 2006) that consists of 19-items. Class collective efficacy was measured by the students’ aggregation of self-efficacy (9-items) and cohesiveness (10-items) in each class. An example of a self-efficacy item is “I am an excellent student in our class,” and cohesiveness “In my class, my classmates and I get along very well.” Each student rated his or her agreement with the items on a 6-point Likert scale ranging from 1 (*strongly disagree*) to 6 (*strongly agree*). In this study, the α coefficients for the total scale, self-efficacy subscale, and cohesiveness subscale were 0.93, 0.91, and 0.94, respectively. In addition, the results of the confirmatory factor analysis were as follows: χ^2^/*df* = 2.13, *p* < 0.001; CFI = 0.92; NNFI = 0.93; RMSEA = 0.060; SRMR = 0.07.

### Analytical Approach

We observed meaningful between-class variance [*F*(27,1006) = 21.93, *p* < 0.001; ICC(1) = 0.17, *p* < 0.01; ICC(2) = 0.87; r_WG(J)_ = 0.92] ranging from 0.74 to 0.98. Considering the between-class variance, it was imperative to test the cross-level interactions (i.e., Hypotheses 4 and 5)—to investigate whether the class collective efficacy and class size moderation of the externalizing behavior-academic engagement relationship (Hofmann and Gavin, 1998).

Because the individuals were nested within classes (multilevel nature of the data), the data in the present study were inherently multilevel, with class size and class collective efficacy being at the class level of analysis, and externalizing behavior and academic engagement being at the individual level of analysis. The most appropriate analytical method is one that takes into account this multilevel data structure. Accordingly, we used hierarchical linear modeling (HLM) to test all hypotheses. We used HLM 6.02 software, wherein Level 1 represents individual data and Level 2 represents aggregated class data. Our hypotheses included cross-level effects from Level 2 (class) to Level 1 (individual), and we followed the steps outlined by Hofmann and Gavin (1998). We report both generalized least squares (GLS) standard errors and more robust standard errors. Considering our Level 2 sample size, we report only the *t*-values based on the more conservative GLS estimates.

## Results

**Table 1** presents the mean, standard deviation, and α coefficient of each variable in this study, as well as the relevant coefficients between variables. As expected, we observed a significant relationship between externalizing behavior and academic engagement (**Table 1**), which provided initial support for Hypothesis 1. Significant correlations were identified between class collective efficacy and academic engagement (*r* = 0.32; Hypothesis 2), whereas no significant correlations were observed between class size and academic engagement (*r* = -0.007; Hypothesis 3). In these correlations, however, the multilevel nature of the data was not taken into account. Thus, we conducted HLM analyses.

**Table 1:** Means, standard deviations, and intercorrelations among study variables.

	*M*	*SD*	1	2	3	4
(1) Class size	36.93	6.31	–			
(2) Class collective efficacy	85.60	10.22	-0.23^∗∗^			
(3) Externalizing behavior	6.40	4.01	-0.02	-0.16^∗∗^		
(4) Academic engagement	52.15	21.14	-0.07	0.32^∗∗^	-0.39^∗∗^	

*N = 1034. Externalizing behavior and academic engagement at the individual level; class size and class collective efficacy at the class level. ^∗∗^*p* < 0.01*.

**Table 2** presents the results of the null models, indicating substantial within- and between-class variance for academic engagement (*σ*^2^ = 372.59, τ_00_ = 75.21). Chi-squared tests revealed that the between-class variance was significant for academic engagement [academic engagement: τ_00_ = 76.56, χ^2^(27) = 274.04, *p* < 0.001]. The intraclass correlation ICC(1) = τ_00_/(*σ*^2^ + τ_00_) for academic engagement was 0.17, signifying that the observed between-class variance accounted for 17% of the total variance associated with academic engagement. This result suggest that 83% of the overall variance is consisted of within-class variation. Specifically, these results suggest that academic engagement varied considerably from class to class.

**Table 2:** Hierarchical linear modeling models and results for Hypotheses 1.

Model equations	γ_00_	γ_10_	*σ*^2^	τ _00_	τ _11_
**Null models**
AE = β _0_*_j_* + r*_ij_*	52.35	–	372.59	75.21^∗∗∗^	


β_0_*_j_* = γ_00_ + u_0_*_j_*					


**Hypothesis 1**
L1: AE = β*_0j_* + β_1_*_j_*(EB) + r*_ij_*	50.29	-1.78^∗∗∗^	316.30	76.56	0.55^∗∗^
L2: β_0_*_j_* = γ_00_ + u_0_*_j_*					
L2: β_1_*_j_* = γ_10_ + u_1_*_j_*					

*L1, Level 1; L2, Level 2; β_0_*_j_* is AE for the individual; γ_00_ is AE for class; σ^2^ = var(r*_ij_*) is individual variance for AE; and τ_00_ = var(u_0_*_j_*) is class variance for AE. ^∗∗^*p* < 0.01, one-tailed; ^∗∗∗^*p* < 0.001, one-tailed*.

To investigate Hypothesis 1, we first conducted random effect regression on student-level data by using HLM. Academic engagement was the outcome variable and externalizing behavior was the predictor variable. HLM revealed that externalizing behavior was significantly negatively related to academic engagement (γ_10_ = -1.78, *p* < 0.001, one-tailed), supporting Hypothesis 1. However, Hypotheses 2 and 3 suggested that class collective efficacy and class size would predict academic engagement. Thus, academic engagement was also the outcome variable, whereas an aggregate of each class’s collective efficacy and class size served as the predictor variables; a cross-level model was subsequently established (**Table 3**, top part). Additionally, we tested the possibility of a significant between-class interaction between class collective efficacy and class size. The results of this first model are outlined as follows: (a) Class collective efficacy (γ_01_ = -0.72, *p* < 0.0001) was a positive predictor of academic engagement; (b) the main effects of class size (γ_02_ = -0.23, *p* = 0.078), as well as the interaction between class collective efficacy and class size (γ_03_ = 0.02, *p* = 0.89), were not significant; and (c) significant variance was observed in the Level 1 slopes relating externalizing behavior to academic engagement (U1 variance 0.49; χ^2^(25) = 52.38, *p* < 0.01).

**Table 3:** Hierarchical linear modeling models and results for Hypotheses 2–5.

Model equations	γ_00_	γ_01_	γ_02_	γ_03_	γ_10_	γ_11_	γ_12_	*σ*^2^	τ _00_	τ_11_
**Hypothesis 2–3**										
L1: AE = β*_0j_* + β_1_*_j_*(EB) + r*_ij_*	52.37	0.72^∗∗^	-0.23		-1.98^∗∗∗^			35.14	33.54	0.49^∗∗^
L2: β_0_*_j_* = γ_00_ + γ_01_(CCE*_j_*) + γ_02_(CS*_j_*) + u_0_*_j_*										
**Hypothesis 4–5**										
L1: AE = β_0_*_j_* + β_1_*_j_*(EB) + r*_ij_*	52.38	0.66^∗∗^	-0.22	0.02	-2.03^∗∗∗^	-0.056^∗∗^	-0.057^∗∗^	34.55	77.69	0.29
L2: *β*_0_*_j_* = γ_00_ + γ_01_ (CCE*_j_*) + γ_02_(CS*_j_*) + γ_03_ (CCE*_j_* × CS*_j_*) + u_0_*_j_*										
L2: β_1_*_j_* = γ_10_ + γ_11_(CCE*_j_*) + γ_12_(CS*_j_*) + u_1_*_j_*										

*CS, class size. ^∗∗^*p* < 0.01, one-tailed; ^∗∗∗^*p* < 0.001, one-tailed*.

**FIGURE 2:**
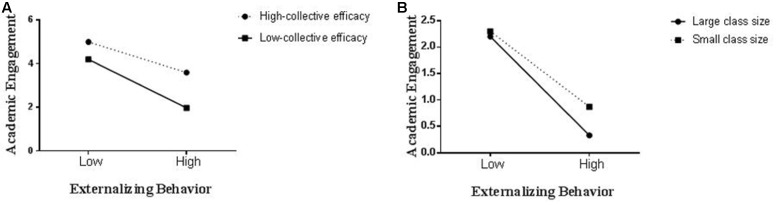
Class collective efficacy **(A)** and class size **(B)** as moderators of the relationship between externalizing behavior and academic engagement.

Then we established a moderation model (**Table 3**, bottom). In this model, class collective efficacy and class size were added as a predictor of the variance in the slopes relating externalizing behavior to academic engagement. The results of this model revealed that the cross-level interaction was significant for (a) class collective efficacy (γ_11_ = -0.056, *p* < 0.001), and (b) class size (γ_11_ = -0.057, *p* < 0.001). These indicate that the relationship between externalizing behavior and academic engagement had statistically significant variations across classes. Additionally, after class collective efficacy and class size were included in the model, the residual variance in the Level 2 slopes was not significant [i.e., U1 variance 0.29; χ^2^(25) = 36.44, *p* = 0.065, *ns*]. Using these two variance components, we calculated that the *R*^2^ value for class collective efficacy and class size was 0.40 [i.e., (0.49 - 0.29)/0.49]. This significant cross-level interaction is shown in **Figure 2**, where the relationship between externalizing behavior and academic engagement are plotted for high and low class collective efficacy and class size (defined as +1 and –1 standard deviation from the mean, respectively; Aiken and West, 1991). The simple slope of the regression of externalizing behavior on academic engagement with high class collective efficacy (simple slope = -1.64), *t*(24) = -6.30, *p* < 0.001, and a small class size (simple slope = -1.21), *t*(24) = -3.24, *p* < 0.01, was significant. With low class collective efficacy (simple slope = -0.06), *t*(24) = -0.09, *p* = 0.76, and a large class size (simple slope = -0.32), *t*(24) = -0.60, *p* = 0.41, the slope was non-significant. Thus, consistent with Hypotheses 4 and 5, externalizing behavior and academic engagement exhibited a weaker negative relationship in a class with high collective efficacy and small size.

The cross-level interaction (**Figure 2**) represents how the within-group relationship between externalizing behavior and academic engagement changes as a function of class collective efficacy (**Figure 2A**) and class size (**Figure 2B**). Accordingly, we investigated the relationship between externalizing behavior and academic engagement within a class as a function of between-class differences in class collective efficacy and class size.

## Discussion

The results of our study are summarized as follows: (a) Academic engagement was significantly negatively related to externalizing behavior; (b) academic engagement was, however, significantly positively related to class collective efficacy; and (c) the relationship between externalizing behavior and academic engagement was moderated by both class collective efficacy and class size, constituting the most notable finding of this investigation. Specifically, for students in classes with high collective efficacy or small size (i.e., ≤30 students), the relationship between externalizing behavior and academic performance was weaker than that for those in a class with low collective efficacy or large size (i.e., ≥43 students).

As expected, externalizing behavior did influence students’ academic engagement. This finding is consistent with those of previous studies (Abikoff et al., 2002; Junod et al., 2006). As described by Baker et al. (2008), “students with externalizing behavior often lack sufficient self-regulation, are academically unprepared and unmotivated, lack time management strategies, and may be tardy and unprepared to make an active and effortful contribution, even to the point of being hostile toward academic engagement.” In addition, high online activity, drinking, smoking, and other delinquent behaviors constitute a large portion of their time, consequently resulting in them having greatly lowered time for academic engagement. Thus, it is imperative for teachers to determine how to facilitate academic engagement and buffer against the disruptive effects of externalizing behavior on academic engagement.

We also determined that class collective efficacy was significantly associated with academic engagement. As described by Bandura (1977), “efficacy beliefs, referring to perceptions of task-specific capabilities, are a key mechanism of behavioral change for individuals.” For example, in a class with high collective efficacy, students perceived more self-efficacy beliefs and they were more likely to persist in academic endeavors. Specifically, “self-efficacy leads to a greater willingness to spend additional energy and effort on completing a task or an assignment and hence to more task involvement and absorption” (Schaufeli and Salanova, 2007; Ouweneel et al., 2011). The rationale is straightforward: For students who believe their actions can make a difference, they would be more likely to exert self-regulation. In addition to self-efficacy, class cohesiveness could have an influence on academic engagement. Specifically, cohesive classes tend to experience more positive emotional states (Kidwell et al., 1997); “when students feel emotionally supported in class, they are more likely to use self-regulatory strategies, such as planning, monitoring, and regulating their thinking, which increase their engagement in classroom tasks” (Patrick et al., 2007). Furthermore, students’ experience of positive emotions could increase self-efficacy, which, in turn, increase self-efficacy–facilitated academic engagement (Schaufeli and Van Rhenen, 2006; Ouweneel et al., 2011).

Class collective efficacy proved to be a beneficial moderator. This may be because students must be active learners; in particular, they need the power of self-regulation (self-overseeing and self-steering their own learning) to become more committed, responsible, and effective learners (Clark, 2012). Specifically, when students got hyperactive, impulsive, and aggressive behaviors, which made them had difficulty shifting or focusing attention, persistence on tasks. Classes with high collective efficacy afforded students more confidence (self-efficacy) for self-regulation and in turn more engagement behaviors followed. However, students in the class with low collective efficacy assumed that they did not possess sufficient capabilities for self-regulation; hence, when they were confused with externalizing behavior, they did nothing. By contrast, the class with high cohesiveness had more positive emotions, which yielded more class resources (e.g., sharing of learning intentions, conducting collaborative learning, and effective discussion) for managing the disruptive effects of externalizing behavior. In addition, positive emotions could yield greater personal resources (e.g., self-efficacy, hope, resilience, and optimism), which further facilitated self-regulated behaviors for academic engagement (Ouweneel et al., 2011).

Notably, class size also proved to be a beneficial moderator. We believe that students might benefit from small classes because more positive teacher–student and student–student interactions are inherent in such classes (Pianta, 1999; Skalická et al., 2015). In the present study, we interpreted the aforementioned interactions with the help of the principles of two main social environment theories. As described by Finn et al. (2003), “students in small classes cannot easily avoid being noticed and it is more difficult than in large classes for teachers to ignore them. Thus, teachers can more easily monitor and respond in a timely manner to externalizing behavior while being more effectively positioned to notice—and reinforce—positive behavior. Additionally, small classes can foster greater cohesiveness, and thus positive relationships, among group members, including the provision and receipt of emotional support, which in turn increases academic engagement.” In such circumstances, academic engagement behaviors may ultimately be considered more acceptable and perhaps normative behaviors (Konstantopoulos, 2009; Stuhlman and Pianta, 2009; Reyes and Brackett, 2012).

Additionally, class size could not directly predict individuals’ academic engagement. This result is consistent with previous studies (Shen and Konstantopoulos, 2017; Yamamori et al., 2017). However, not all class size studies have indicated that class size could not predict individuals’ learning behaviors. Hanushek (1999) reviewed 276 studies and determined that 14% of the studies indicated that smaller class sizes contribute to more favorable learning behaviors, 14% indicated the opposite, and 72% indicated that smaller class sizes contribute to neither fewer nor more learning behaviors. Some studies have suggested that the relationship between class size and academic engagement is country and context specific (Skalická et al., 2015; Yamamori et al., 2017). For example, a recent study identified a positive significant relationship between class size and reading achievement in Germany and a negative significant relationship between class size and reading achievement in Romania (across years); however, the relationship between class size and reading achievement was non-significant across eight European countries (Yamamori et al., 2017).

### Implications for Teachers

The present study provided possible implications for teachers to facilitate students’ academic engagement and buffer against the disruptive effects of externalizing behavior on academic engagement. Bandura (1977) suggested that “students are active self-regulated learners, and have some metacognitive and motivational qualities with which to regulate their behaviors while the classroom setting either facilitates or frustrates the acquisition and use of self-regulatory characteristics.” For example, students with externalizing behavior were blamed for their poor grades, and this could reduce self-regulatory behaviors. Because students with externalizing behavior were confused with self-regulation difficulties, they needed more confidence from class settings to facilitate their self-regulation, and blame caused them be unready to self-regulate, and even hostile to learning. However, this study determined that classes with high collective efficacy increased the buffer against the disruptive effects of externalizing behavior on academic engagement. This indicates that positive psychological interventions about boosting academic engagement are more effective than preventing the negative effects of externalizing behavior. Thus, it is imperative for teachers to foster their classes with positive psychological interventions such as by increasing collective efficacy.

The present study also provides teachers with two practices (self-efficacy and cohesiveness) for raising collective efficacy in class. The first practice entails matching the task demands to meet a student’s skill level. The challenge-skill balance model posits that if the demands of a situation or task exceed the skills and coping resources of a person, that person experiences stress and loses confidence. However, if the task is insufficiently challenging, the person experiences boredom and loses interest in learning. Only optimally challenging tasks enable people to feel a sense of competence and high self-efficacy (Deci and Ryan, 2000). The second practice involves periodical collective counseling and collective activities. These activities can facilitate students to exert positive interactions such as respecting, encouraging, and helping each other, which can help the class stay in positive emotional and highly cohesiveness states (Jacobs, 2010).

In summary, the class is considered one of the most crucial developmental systems in the lives of students after that of the immediate family (Bronfenbrenner and Morris, 2006). The positive influence of class settings can be used to increase students’ academic engagement. In addition, this study highlights small class instruction as having protective effects on academic engagement. Because of the absence of regulations stipulating permitted class sizes in China, the present study calls attention to the potential utility of regulating junior middle school class sizes in China, or even of eventually reducing the currently regulated maximum class sizes in other countries.

### Limitations and Future Directions

This study provides teachers with a new perspective for intervening in the relationships of externalizing behavior and academic engagement. However, this research is subject to certain limitations. First, subjects were students from a junior middle school, and the conclusions are not generalizable to other students. Future research should further explore the functions of class settings in other types of schools such as elementary and senior middle schools. Second, this was a cross-sectional study, and although we theorized in terms of causality, we could hardly provide a causal inference, and common method issues may have affected the lower level relationships. Therefore, more experimental studies are required. In addition, future research could perform longitudinal studies, particularly to study the outcomes of a student transferring from one class to another. How the student’s externalizing behavior changes and how such changes affect academic engagement under the influence of different class settings can also be explored. Third, China has recently undertaken reforms making its educational system more similar to those in the United States and other countries, particularly introducing “shift classes,” where students sometimes have a fixed class, but at other times do not—a revolutionary reform of China’s traditional education mode—a result of which is that the function of class setting may change. In this study, all students were from a fixed class rather than shift classes. Therefore, future studies should undertake research on the students of shift classes.

## Author Contributions

YT wrote the manuscript; collected the data; analyzed the data under the supervision of FG. YB collected the data; analyzed the data. PH collected the data; analyzed the data. FG wrote the manuscript; designed the study. PW collected the data; analyzed the data; wrote the manuscript.

## Conflict of Interest Statement

The authors declare that the research was conducted in the absence of any commercial or financial relationships that could be construed as a potential conflict of interest.
